# Mycoviruses in Fungi: Carcinogenesis of Fungal Agents May Not Always Be Mycotoxin Related

**DOI:** 10.3390/jof9030368

**Published:** 2023-03-17

**Authors:** Cameron K. Tebbi

**Affiliations:** Children’s Cancer Research Group Laboratory, 13719 North Nebraska Avenue, Suite #108, Tampa, FL 33613-3305, USA; ctebbi@childrenscancerresearchgrouplaboratory.org

**Keywords:** cancer, leukemia, etiology, mycoviruses, mycotoxins, aflatoxin, viruses, carcinogenesis, leukemogenesis, fungi, parasites

## Abstract

Certain viruses have been found to induce diverse biological pathways to carcinogenesis, evidenced by the presence of viral gene products in some tumors. Despite the fact that many fungal agents contain mycoviruses, until recently, their possible direct effects on human health, including carcinogenesis and leukemogenesis, had not been explored. In this regard, most studies of fungal agents have rightly concentrated on their mycotoxin formation and effects. Recently, the direct role of yeasts and fungi in the etiology of cancers, including leukemia, have been investigated. While greater attention has been placed on the carcinogenic effects of *Candida*, the role of filamentous fungi in carcinogenesis has also been explored. Recent findings from studies using the enzyme-linked immunosorbent assay (ELISA) technique indicate that the plasma of patients with acute lymphoblastic leukemia (ALL) uniformly contains antibodies for a certain mycovirus-containing *Aspergillus flavus*, while controls are negative. The exposure of mononuclear leukocytes from patients with ALL in full remission, and long-term survivors, to the product of this organism was reported to result in the re-development of typical genetics and cell surface phenotypes characteristic of active ALL. Mycoviruses are known to be able to significantly alter the biological characteristics and functions of their host. The possible carcinogenic and leukemogenic role of mycoviruses, with and without their host, needs to be further investigated.

## 1. Introduction

All individuals are routinely exposed to a variety of fungal organisms, for the most part without any detectable significant adverse effects. However, this is not universal and varies based on health status, immunity, existence of other disorders, and type of the organism involved. For example, exposure to *Aspergillus* spores routinely occurs in healthy populations without any obvious clinically detectable effects. However, the same exposure can result in serious pathogenic effects in individuals with certain underlying diseases such as cancer or immune deficiency disorders. Until recently, the major known direct pathogenic effects of *Aspergillus* species have included allergies, toxicities, and a variety of infections [1,2,3,4,5,6,7,8,9,10,11,12,13,14,15,16,17,18,19,20,21,22,23,24,25,26,27,28,29,30,31,32,33,34,35,36,37,38,39,40,41,42,43,44,45,46,47,48,49,50,51,52,53,54,55,56,57,58,59,60,61] (Table 1). A substantial amount of data, however, has been accumulated, indicating that those working in the occupations with a high degree of fungal exposure generally have a higher rate of cancer [5,62,63,64,65]. In contrast, while somewhat controversial [66,67,68,69,70,71,72], individuals with allergy-related diseases and asthma have been reported to generally have a lower rate of cancer, including leukemia and a variety of solid tumors, as compared to the general population [73,74,75,76,77,78,79,80,81,82,83,84]. Some epidemiological data indicates potential roles for IgE, allergy, and atopy in protecting against certain tumors [85,86]. An increased cancer risk in association with IgE immunodeficiency has also been reported [87,88]. Significant information regarding the inverse association between atopic conditions and glioma has been accumulated [89,90].

Some microorganisms are known to have the ability to induce tumor initiation and progression, directly through their effects on the cells, or indirectly by their effects on the immune system. Several studies have correlated the possible involvement and association of fungal species, particularly *Candida*, with the development and progression of various types of cancer. While most attention has been directed to yeasts in the so-called blastomycete theory of cancer, more recently, other mechanisms, including the possible role of mycoviruses in fungal organisms, have been suggested [91,92,93,94,95,96,97,98,99]. In the recent past, it has been shown that the in vitro exposure of mononuclear cells from individuals who have a history of acute lymphoblastic leukemia (ALL) and are in full remission, without any evidence of the disease, including long-term survivors, to a certain mycovirus-containing *Aspergillus flavus* (MCAF) results in the re-development of cell surface phenotypes and genetic markers characteristic of active ALL [91]. Such exposure in controls did not induce any changes [91]. If this is due to the certain genetic or epigenetic background in the ALL patients is not clear. In a related study, unlike controls, patients with ALL were found to have antibodies for the products of MCAF [92]. It is of interest that the mycovirus-containing *Aspergillus flavus* used for these studies was isolated from the home of a patient with ALL and the organism did not produce any aflatoxin [91,92]. A well-known theory for the etiology of ALL in the pediatric age group suggests that the development of this disease is due to a combination of genetic mutations and exposure to infections [100]. This so-called “two-hit” model postulates that ALL evolves in two discrete steps. The first step is in utero initiation by fusion gene formation or hyperdiploidy, which generates a pre-leukemic clone. The second step, which is proposed to only occur in a very small sub-population of the predisposed, is suggested to be exposure to infections and post-natal acquisition of secondary genetic changes that drives conversion to overt ALL. It is postulated that exposure to infections earlier in life are protective, but in their absence, infections later in life trigger the critical secondary mutations [100]. It is suggested that the risk can be further modified by other inherited genetics, and possibly diet, as well as chance. While a number of predisposing genetic factors have been identified, until recent reports [91,92], no certain infectious agents had been proposed. Based on the recent findings, it is postulated that exposure to mycovirus-containing *Aspergillus flavus* could possibly be one of the post-natal infections that can trigger the second step in the developmental process of ALL [91].

A number of studies have explored the role of viral, bacterial, and fungal organisms in the etiology of a variety of cancers. In the past, many investigations have concentrated on the correlation of viruses with the development of malignant disorders. While such a correlation has been made for many viral agents, less attention has been paid to the fungal organisms, including those containing mycoviruses. Typically, most studies have been based on the statistical relation of the frequency of exposure to a given agent, versus the development of a certain cancer. For example, there is an increased rate of cancer and leukemia [5] in individuals likely be exposed to fungal agents, such as agricultural workers [101]. More recently, experiments have reported direct evidence of some organism’s involvement in the development of certain cancers. For example, some experiments have revealed that DNA specific to the human papilloma virus (HPV) is integrated into the host cell genome. This virus is known to be associated with cervical, anal, penile, vulvar, vaginal, oropharyngeal, and head and neck cancers [102]. HPV type 16 and 18 viral DNAs have been found in cervical cancers and two viral dominant oncogenic genes, E6 and E7, are consistently expressed in HPV-positive malignancies and HPV-infected cancer cells. These oncogenes are known to be associated with the malignant transformation of cells and the alteration of the immune system, causing disruption of natural tumor suppressor pathways, culminating in the proliferation of cervical carcinoma cells [103].

Fungi have a worldwide distribution and virtually can be detected in any environment [104,105,106,107,108,109,110,111,112,113,114]. For example, *Aspergilli* are found in soil, water, outdoor and indoor, and may produce numerous conidia which can disperse via air movement and possibly insects [104,105,106]. While there are seasonal variations, there is a significant amount of spore and diversity of fungi in air particulate matter [107]. The optimum temperature for the growth of these organisms has a wide range, with significant growth occurring in temperatures ranging from approximately 15 to 30 °C [108,109,110,111]. Lately, the direct effects of yeast and fungi in the etiology of cancers, including leukemia, have also been further explored. Greater attention has been placed on the so called “blastomycete theory of cancer” and the relation of yeasts, especially the *Candida* species, to carcinogenesis [112]. To a lesser extent, the role of filamentous fungi has also been evaluated. Among the fungal agents, *Aspergillus* and *Candida* species are the most investigated for their possible role in carcinogenesis. *Aspergilli*, due to their widespread distribution and production of mycotoxins, are known for their potential to cause cancer [93,112]. These have been classified and divided into several groups, each with distinct biological and molecular characteristics [93,115,116,117,118]. As noted above, *Aspergilli* are known to produce a variety of allergic disorders [1,2,3,5], toxicities, and infections [17,22,23,24,54,55,56,57,58,61]. *Aspergillus* can be associated with tissue damage, burn, keratitis and endophthalmitis, or post operative infection [13,16,119,120,121,122,123,124,125,126,127]. Empyema and pleural aspergillosis, as well as osteomyelitis, can occur, especially in individuals with reduced immunity. The association of *Aspergillus* infections with a foreign body, including peritoneal dialysis catheters and intravenous lines have been reported. Abscesses in various organs and systems, including skin, subcutaneous tissues, sinuses, oropharynx, lungs, brain, and other organs and systems can occur. In many infection entities, *Aspergillus fumigatus* or *A. flavus* are often involved [13,16,119,120,121,122,123,124,125,126,127]. The effects of mycotoxins produced by fungal agents have been long recognized [113,114].

Normally, *Candida* is located on skin, most of mucosal surfaces, mouth, gastrointestinal tract, and vagina without any detrimental effects. However, *Candida* species in general, and *Candida albicans* in particular, have been found to be associated with the development of certain cancers, including oral, esophageal, and gastrointestinal neoplasms. Some of these carcinogenic effects are through the production of specific hydrolytic enzymes metabolizing ethanol to acetaldehyde (ACH). Acetaldehyde is metabolized from ethanol by alcohol dehydrogenases (ADH). ADH is a cell wall protein necessary for the growth of fungi, its metabolism [128], and interaction with host cell proteins to initiate an immune response [128,129,130]. Mutations within host DNA degrade protein molecules and impair their functions which are essential for normal cellular activities and division [131]. ACH, which is a group 1 human carcinogen, can induce the production of pro-inflammatory cytokines and mediators [132,133,134], causing cellular oxidative stress and damage [134], inducing the formation of covalent adducts in protein or DNA residues [131], DNA cross-linking, or chromosomal aberrations [135,136]. The aberrations described can potentially lead to tumor development and progression [95,137]. There is evidence that pathologically, *C. albicans* can increase the risk of carcinogenesis and metastasis through several other mechanisms, including inflammation, the production of carcinogenic byproducts, the induction of the Th17 response, and molecular mimicry [137,138,139,140,141,142,143,144,145,146]. In addition to several clinical reports associating *Candida* spp. to carcinogenesis, there are a number of biomolecular findings indicating its ability to cause dysplasia and malignant neoformation in oral epithelium. *Candida* can produce carcinogens, such as N-nitrosobenzylmethylamine, resulting in the development of malignant disorders including oral cancer [138,139,140,141]. The development of pancreatic cancer has also been attributed to inflammation and immune activation due to an increased nitrosamine exposure. Some of the mechanisms of actions suggested for the carcinogenic effects of *Candida* include over-expression of P53, *Ki*-*67* labeling index, and Prostaglandin-endoperoxide synthase 2 (COX-2), promoting the production of acid aspartyl-proteinase. Other effects include immune-related mechanisms induced by up-regulation in proinflammatory cytokines such as interleukin (IL)-1α, IL-1β, IL-6, IL-8, and IL-18, tumor necrosis factor (TNF)-α, IFN-b [138,142,143], and the production of carcinogenic acetaldehyde [137,144,146] and candidalysin, which is a cytolytic toxin [146].

### Mycotoxins

More than 350 types of mycotoxins are found in animal feed but the most important are aflatoxin, ochratoxin, fumonisin, and zearalenone [147,148,149,150,151,152,153,154,155,156,157,158]. The pathological condition induced by any mycotoxin depends on sex, immune status, type of mycotoxin, duration, and the amount of mycotoxin. Mycotoxins are responsible for the suppression of the quality of the poultry industry. According to the Food and Agriculture Organization (FAO), 25% of cereal grains are found to be affected by mycotoxins. Aflatoxin is one of the most important mycotoxins produced by *A. flavus* and *A. parasiticus*. More than 20 types of aflatoxins are found. The most common derivatives of aflatoxin are B1, B2, M1, and M2. B1 is the most potent carcinogenic mycotoxin and M1 is the most common in milk.

The carcinogenesis and leukemogenesis of many fungal species, in animals and humans, have traditionally been attributed to their production of mycotoxin. Mycotoxins are toxic secondary metabolites that are produced by fungal species, particularly those of filamentous fungi which often grow on plant-based agricultural products. The fungal growth occurs prior to the harvest of the crops and during their storage. Mycotoxins can be found in peanuts, grains, corn, millet, sesame seeds, wheat, and animal-derived foods such as milk, eggs, meat, and other commodities, and are highly toxic to humans and animals. A single fungal species can potentially produce several mycotoxins [148,150]. The hepatotoxicity and hepatocarcinogenic effects of fungal agents secondary to the production of mycotoxins in general, and aflatoxin in particular, are well recognized [91,93]. Some mycotoxins affect DNA replication, and therefore, can have mutagenic or teratogenic effects. Exposure to mycotoxins can result in the impairment of metabolic, nutritional, endocrine, immunological, hepatic, reproductive, and other systems. The four basic toxicities of mycotoxins are acute, chronic, mutagenic, and teratogenic effects. Common acute mycotoxin poisoning effects includes deterioration of liver or kidney function, which can potentially lead to death. Some mycotoxins interfere with protein synthesis, causing disorders ranging from skin sensitivity or necrosis to immunodeficiency, depending on the dose exposed. Mycotoxins are neurotoxic, producing symptoms ranging from trembling to brain damage. An example of the major biotoxins produced by *Aspergillus* species are summarized in Table 2. Aflatoxins, produced by *Aspergillus* spp., are one of the highly toxic secondary metabolites derived from polyketides. These are known to induce acute intoxication, fulminant hepatic failure, and rhabdomyolysis. Chronic exposure to this toxin can result in cirrhosis of the liver which may lead to hepatocellular and gall bladder carcinoma. Other effects of aflatoxins on human health include disorder of lipid metabolism, depression of protein and enzyme synthesis, and reduced production of hemoglobin and response to vaccines [149,150]. The mycotoxins produced by *Penicillium* and *Fusarium* species have adverse effects on heath, including infertility in males and females, destructive effects on the fetus, impairment of growth and development in children, and undesirable health outcomes in various stages of life. These agents can hamper the division and differentiation of the gametes which can result in infertility due to interference with spermatogenesis. Zearalenone has been linked to precocious puberty in females. In animal models, exposure to mycotoxins can promote adverse effects on spermatozoa, Sertoli and Leydig cell function, oocyte maturation, and uterine and ovarian development and function. These agents have the potential to damage the sex organs. Mycotoxins may disturb the endocrine system and alter steroid hormone homeostasis, resulting in subfertility or infertility. These can exert oxidative stress causing sperm DNA damage and reduced fertilization [151,152,153,154,155]. Based on animal studies, mycotoxins can increase the possibility of stillbirth and can pass through the mother’s milk and affect the health of infants [156,157,158,159]. Since mycotoxins can negatively alter cell division, they can affect the fetus and decrease the growth and development of children. Neural tube defects in fetuses have been reported. Adverse effects on fetuses and children include abnormal neural development, causing cognitive disability. In addition, these toxins may cause decreased gastrointestinal absorption resulting in malnutrition and reduced growth [160,161,162,163,164,165].

## 2. Metabolism of Aflatoxins

Aflatoxins are furanocoumarins which are produced by various strains of *Aspergillus* species and produce various toxicities in animals and humans [166,167,168,169,170,171]. The toxicity and mechanism of action of aflatoxins have been explored [166,167,168,169,170,171]. The carcinogenic and mutagenic activities of aflatoxins are largely attributed to their lactone and difuran rings. Aflatoxins have a furanocoumarin chemical structure, with over 18 types chemically identified. Following ingestion, AFB_1_ is metabolized to form AFB_1_-8,9-epoxide, which binds to DNA and forms AFB_1_-guanine adducts. There are significant individual and age-related differences in the metabolism of AFB_1_, resulting in variation noted in its toxicity. In vitro metabolism studies reveal that reduction of AFB_1_ results in the production of aflatoxicol (AFL), which its hydroxylation produces AFM_1_, its hydration generates AFB_2a_ and its epoxidation AFB_1_-2,3-epoxide. Of these, epoxide is the most reactive, and is believed to be responsible for the acute and chronic toxicity of AFB. Aflatoxicol can pass through the placenta and damage the fetus. AFB1 is known to be metabolized in the liver by the cytochrome P450 enzyme system (CYPs). Aflatoxin B1-8,9-epoxide (AFB0), which has *exo* and *endo* isomers, is a carcinogenic derivate of this toxin. The CYP3A4 and CYP1A2 derivates are primarily responsible for the aflatoxin biotransformation, and the *exo* isomer formed. Studies in birds indicate that CYP2A6 and, to a lesser extent, CYP1A1 are involved in the bioactivation of AFB_1_; into AFBO. Regarding DNA, AFB0 binds covalently to the N_7_ position on guanine, and forms an AFB1-N_7_-guanine adduct. The *endo* isomer has lower affinity than the *exo*; therefore, AFB1-*exo*-8,9-epoxide is likely the major carcinogenic metabolite [158,171]. The production of the various mycotoxins varies based on numerous factors. For example, fungal organisms overwinter as either resistant structures called sclerotia or as mycelium. The difference in the pattern of sclerotia production is associated with different aflatoxin production. An example is that the S strain of *Aspergillus flavus* produces numerous but smaller sclerotia, while the L strain generates fewer but larger sclerotia. The products of these subgroups vary significantly. The S strain isolates, designated SB, make only B aflatoxins while those termed SBG produce B and G aflatoxins. It is suggested that these differences may represent a taxon different from *Aspergillus flavus.*

Some strains of *Aspergillus flavus* do or do not produce aflatoxins B1 and/or B2. Other toxins which may be produced by this organism include cyclopiazonic acid, kojic acid, sterigmatocystin, bnitropropionic acid, aflatrem, aspertoxin, aspergillic acid, and gliotoxin. In addition, *Aspergillus flavus* can potentially produce other secondary metabolites including versicolorin A dihydroxyaflavinine, paspalinine, and indole. Aflatoxins, which are the most potent hepatocarcinogenic agents, are known to be produced by a variety of *Aspergillus* species, predominantly *A. flavus*, *nomius*, and *parasiticus*. Of sixteen structurally related toxins, aflatoxins B1, B2, G1, and G2 are of the most concern [166,167]. The metabolites produced by the hepatic metabolism of aflatoxins are responsible for most of their toxicity. Aflatoxin B1 (AFB1) has the most carcinogenic potential, and its carcinogenicity is classified as group 1 by the International Agency for Research on Cancer (IARC). Exposure to the aflatoxin metabolites results in acute liver damage. Should this be continued, it has a high potential for carcinogenesis due to the damage to DNA through adduct formation and interference with protein metabolism [168]. In pregnant animal models, exposure to AFB1 leads to genotoxic changes which predisposes the offspring to morphological abnormalities, behavioral alterations, reproductive disturbances, cancer, and early death in adult life [170,171]. In humans and animals, the signs and symptoms of aflatoxin toxicity depends on the level and duration of exposure, age, gender, health status, concurrent exposure to other toxins, and a number of other variables. Generally, adults have a higher tolerance for aflatoxin and rarely succumb to acute aflatoxicosis. In contrast, children are less tolerant and their exposure results in stunted growth and delayed development. The latter is common in many developing countries [172,173,174]. In general, acute aflatoxicosis due to the ingestion or inhalation of high doses of AFB1 results in acute poisoning. These toxins can be transmitted to the fetus through the placenta, and to infants via breast milk [110,111,148,175,176,177,178,179,180,181,182,183]. Severe damage to the internal organs and systems including liver, kidneys, heart, and the hemopoietic and immune system, along with bleeding, can result in death. For survivors, long-term complications include organ and system failures and carcinogenesis. Post exposure, free AFB1 is present only for a short period of time in the blood. Such exposure can be detected through the measurement of the metabolites of AFB1 including aflatoxin-albumin, aflatoxin M1 (AFM1), aflatoxin P1 (AFP1), aflatoxin Q1 (AFQ1), AFB-N7 guanine, and aflatoxicol (AFL), in blood and biological fluids. In the first 24 h post exposure, the measurement of the breakdown products of AFB1, including AFB_1_-guanine, in the urine may reflect exposure to this mycotoxin. Measurement of the AFB_1_-albumin adduct level in the serum provides a more integrated measure of the longer-term exposure. AFM1 is classified as agent 2B by IARC for its carcinogenic potential. Metabolites of aflatoxins are present in the tissues, urine, feces, and milk [184,185,186,187,188,189,190]. The latter is of importance in infants during breastfeeding because of its effects in infants. Likewise, commercially available milk collected from animals fed with various contaminated agricultural commodities may contain this agent. Various metabolites of mycotoxins can be measured in blood, urine, stool, milk, etc. [184,185,186,187,188,189,190]. AFM1 can be utilized as a measurable biomarker in the urine. Immunotoxins with small molecules fail to induce any response in the human immune system. Therefore, a major potential danger of exposure to mycotoxins in the diet is the human inability to detect them biologically [2].

Another product of AFB1 is the AFB-N7-guanine biomarker which indicates prolonged exposure to this toxin. DNA alkylation or adduct formation is at nucleophilic sites in DNA, including the N7-position of guanine. The N7-guanine adducts are considered non-promutagenic. These are chemically unstable, since the N7-position does not participate in a Watson–Crick base pairing. The N7-guanine adducts have been shown to convert to ring opened lesions (FAPy) which have much more mutagenic potential, persist longer in the body, and have higher mutagenic potency [191]. A variant with a greater carcinogenic potential is fumonisin B1 (FB1), which is classified by the IARC as a 2B product. This is predominantly produced by *Fusarium verticillioides* and *F. proliferatum* and contaminates maize and maize-based foods. FB1 inhibits ceramide synthase and interrupts sphingolipid synthesis via the inhibition of sphingosine-N-acetyltransferase, resulting in oxidative stress, the alteration of DNA methylation, and modulation of autophagy, and results in stress to the endoplasmic reticulum, leading to the reduced production of sphingolipids and the accumulation of sphinganine (Sa) and sphingosine (So). The result is the non-genotoxic mechanism underlying its toxicological and carcinogenic effects. While its effect on human health as yet is not fully discovered, in populations consuming large amounts of contaminated maize-based foods, epidemiological and experimental evidence points to this being a risk factor for esophageal cancer and neural tube defects. In animals, fumonisins can cause leukoencephalomalacia in horses, pulmonary edema in swine, and hepatotoxicity and nephrotoxicity in rats [192,193,194,195].

While most studies focus on a single mycotoxin and its effects on human health, animal studies reveal a complex and possibly additive, synergistic, or antagonistic effect [196,197,198,199]. The association of aflatoxins classified as group1 by the IARC, including aflatoxin B1 (AFB1), aflatoxin B2 (AFB2), aflatoxin G1 (AFG1), aflatoxin G2 (AFG2), and aflatoxin M1 (AFM1), with liver cancer is well documented. Various malignant disorders due to rice and cereal contamination with AFB1, including breast, cervical and esophageal cancers, have been reported [200,201,202,203,204,205,206,207]. A significant amount of data regarding carcinogenicity of mycotoxins, alone or in conjunction with unrelated viruses are available; however, the possible effects of mycoviruses singularly or in combination with their fungal host has not been fully explored.

## 3. Mycoviruses and Cancer

For several decades, viruses affecting fungal organisms, known as mycoviruses, have been known to exist [208,209]. However, except for occasional reports, their human pathogenicity and possible role in health has not been fully evaluated. It is estimated that from 30 to 80% of all fungal species, predominantly endophytic fungi, contain mycoviruses. The existence of mycoviruses in *Aspergillus* species is well recognized. The modulation of fungal toxins such as the loss of aflatoxin production in *A. flavus* infected with mycovirus has been reported [210,211]. Mycoviruses possess various forms of viral genomes which include double-stranded RNA (dsRNA), single-stranded RNA (ssRNA), and single-stranded DNA (ssDNA). Currently, the International Committee for the Taxonomy of Viruses (ICTV) records 17 taxa, 16 families, and one genus that does not belong to a family. While most mycoviruses have ds RNA linear genomes, positive-sense ss RNA linear genomes including reverse transcribing RNA linear genomes, negative-sense ssRNA linear genomes, or ssDNA circular genomes also exist [212]. Of these, dsRNA segments most commonly affect fungal organisms. Taxonomically, the fungal dsRNA viruses are classified into seven families which include *Endornaviridae*, *Chrysoviridae*, *Megabirnaviridae*, *Quadriviridae*, *Partitiviridae*, *Reoviridae*, and *Totiviridae*. The transmission of mycoviruses occurs vertically during cell division, forming asexual and sexual spores called sporogenesis, and/or horizontally via mating or hyphal anastomosis through cytoplasmic exchange, and not during the extracellular phase of the viral life cycle. The latter, however has been disputed [212]. Some viruses have a unique self-protective or aggressive ability, producing defensive substances. These products can have a growth-inhibitory activity against several bacterial and fungal species. The term ‘killer strains’ describes yeast and fungal species that can produce ‘killer toxins’ with antimycotic activity for lethal function or self-protection. This killer phenotype is usually associated with double-stranded (ds)RNA mycoviruses and linear dsDNA plasmids. It can also be chromosomally encoded. For example, viruses of the family *Totiviridae* have a unique ability to produce a killer toxin which is capable of lysing susceptible neighboring strains, while they themselves remain immune to the toxin. Four killer toxins, i.e., K1, K2, K28, and Klus, have been reported.

Some dsRNA mycovirus-containing fungal agents have been shown to alter the expression of genes involved in the ribosomal synthesis and programmed cell death of the fungal host. Mycoviruses affecting a human pathogen may also have an effect on the infected individual. For example, *Malassezia* species produce various skin diseases including dandruff, seborrheic dermatitis, and atopic dermatitis. In one study, this organism was found to contain MrV40 mycovirus, which belongs to the family *Totiviridae*. In a reported study, the viral nucleic acid from MrV40 had induced a Toll-like receptor 3 (TLR3)-mediated inflammatory immune response in the bone-marrow-derived dendritic cells. This finding may indicate a role for the included mycovirus in the pathogenicity of *Malassezia* [213,214].

Mycoviruses are known to be able to alter their fungal host’s phenotype, including but not limited to pigmentation, morphology, sexual and asexual sporulation, the production of toxins, and growth. As noted before, the loss of aflatoxin production in *A. flavus* infected with mycovirus has been reported [210,211]. If these organisms can exert any changes in humans or animals infected with mycovirus-containing fungi has not, as yet, been significantly explored. Viral dsRNA is recognized by Toll-like receptor 3 (TLR-3) and several cytosolic sensors and can provoke interferon production in a TLR-3 dependent or independent fashion [215]. An increased rate of cancer in occupations with higher rate of exposure to fungi, such as agricultural and construction workers, have been found [5,101]. Individuals with allergies have been reported to have a decreased risk of certain cancers compared with the general population. In allergic individuals, lower rate of glioma, laryngeal, esophageal, oral, pancreatic, gastric, colorectal, uterine body cancers, and non-Hodgkin lymphoma have been reported. Reports regarding leukemia, thyroid, lung, melanoma, and breast cancer in this group are conflicting. An increased risk of bladder cancer, lymphoma, myeloma, and prostate cancer in individuals with allergies is reported [68,84,101,215]. It is not clear if in those individuals with allergies and decreased rates of cancer, their allergens include fungi. On the other hand, those with greater exposure to fungi have a higher rate of this disorder. As noted before, patients with acute lymphoblastic leukemia were found to have antibodies to a certain mycovirus-containing *Aspergillus flavus* and the exposure of mononuclear blood cells from patients with ALL in full remission to its products resulted in the redevelopment of genetic and cell surface phenotypes characteristic of ALL [91,92]. Based on these findings, it has been postulated that this organism may potentially have a correlation with leukemogenesis [91,92]. Research regarding mycovirus-containing organisms and cancer may have etiological value.

## 4. Conclusions

The possible role of various organisms in carcinogenesis and leukemogenesis has been suspected. While the role of viral agents in the development and progress of a variety of cancers has often been the subject of these investigations, the carcinogenic effects of fungal agents have also been explored. Until recently, the latter has been mostly concentrated on the contamination effects of mycotoxins. These effects result in major toxicities which are of health and commercial concern. Demonstration of the effects of various viral agents in carcinogenesis is exemplified by cervical carcinoma. Experiments reveal that DNA specific to the human papilloma virus is integrated into the host cell genome, and viral oncoproteins E6 and E7 consequently cause the disruption of natural tumor suppressor pathways, culminating in the proliferation of cervical carcinoma cells. Mycoviruses have been shown to alter the biology of their fungal host, such as the secession of aflatoxin production in *Aspergillus* spp. as well as the expression of genes involved in ribosomal synthesis and programmed cell death in several species. The effects of mycoviruses alone or in conjunction with their fungal host in human health is poorly evaluated. Recent studies reveal that patients with acute lymphoblastic leukemia in full remission, and long-term survivors, uniformly have antibodies to a certain mycovirus-containing *A. flavus*. Furthermore, exposure of the mononuclear leukocytes from these patients to the products of the above organism results in the redevelopment of genetic and cell surface phenotypes characteristic of active acute lymphoblastic leukemia pointing to the possible cause and effect relationship. The role of mycoviruses, with and without their hosts in human disorders, particularly in carcinogenesis and leukemogenesis, needs to be explored.

## Figures and Tables

**Table 1:** Major non-mycotoxin-related pathogenic effects of *Aspergillus* species.

Allergies [1,2,3,4,5]
Cutaneous infections [6,7,8],
Rhinosinusitis [9,10,11],
Wound and craniocerebral infections [12,13,14,15,16],
Keratitis [17,18,19,20,21],
Chronic granulomatous sinusitis [22,23,24,25],
Neurotoxicity and meningitis [26,27,28],
Scleritis [29,30,31],
Endophthalmitis [32,33,34],
Otomycosis [35,36,37],
Pericarditis [38,39,40],
Endocarditis [41,42,43],
Mediastinitis [44,45,46],
Osteoarticular disorders [47,48,49,50],
Osteomyelitis [51,52,53],
Urinary tract infections [54,55,56],
Bronchopulmonary [57,58,59]
Local and generalized infections [5,61]

**Table 2:** Major biotoxins produced by *Aspergillus* species.

Type of Mycotoxin	Example of Producing Species
Aflatoxins (AFB1, AFB2) *A. flavus* and *A. parasiticus*	(AFG1, AFG2, AFM1) *A. parasiticus*
Ochratoxins *A. carbonarius*, *A. ochraceus*, and *A. niger*	Patulin *A. clavatus*
Citrinin *A. ochraceus* and *A. terreus*	Aflatrem *A*. *aculeatus*
Secalonic acids *A. aculeatus* and *japonicus*	Cyclopiazonic acid *A. flavus* and *A. oryzae*
Terrein *A. terreus*	Sterigmatocystin *A. versicolor*, *A. nidulans*, and *A. sydowii*
Gliotoxin *A. fumigatus*	*Fumonisins A. welwitschiae* and *A. niger*
